# The Merits of Unconscious Thought in Rule Detection

**DOI:** 10.1371/journal.pone.0106557

**Published:** 2014-08-29

**Authors:** Jiansheng Li, Yawen Zhu, Yang Yang

**Affiliations:** Department of Psychology, Northwest Normal University, Lanzhou, P. R. China; Utrecht University, Netherlands

## Abstract

According to unconscious thought theory (UTT), unconscious thought is more adept at complex decision-making than is conscious thought. Related research has mainly focused on the complexity of decision-making tasks as determined by the amount of information provided. However, the complexity of the rules generating this information also influences decision making. Therefore, we examined whether unconscious thought facilitates the detection of rules during a complex decision-making task. Participants were presented with two types of letter strings. One type matched a grammatical rule, while the other did not. Participants were then divided into three groups according to whether they made decisions using conscious thought, unconscious thought, or immediate decision. The results demonstrated that the unconscious thought group was more accurate in identifying letter strings that conformed to the grammatical rule than were the conscious thought and immediate decision groups. Moreover, performance of the conscious thought and immediate decision groups was similar. We conclude that unconscious thought facilitates the detection of complex rules, which is consistent with UTT.

## Introduction

When people face important or complicated decisions, they usually confront such situations using conscious thought. However, an increasing amount of research has revealed that certain complex decisions made unconsciously yield higher quality results than those made using conscious deliberation [1]–[4]. On the basis of such findings, Dijksterhuis and Nordgren (2006) proposed unconscious thought theory (UTT) and suggested that people employ two types of decision-making processes: conscious thought (CT) and unconscious thought (UT). CT refers to “object-relevant or task-relevant cognitive or affective thought processes that occur while the object or task is the focus of one's conscious attention,” whereas UT refers to “object-relevant or task-relevant cognitive or affective thought processes that occur while conscious attention is directed elsewhere” [5].

A core feature of UTT is that CT is used to follow strict rules for the purpose of making precise computations, while UT is used to make rough estimates [5]. However, the processing of rules is not limited to the act of following them but also includes conforming to those rules [6]. For example, although an apple cannot actively follow the rule to fall downward, it will conform to the rule of dropping. One important aspect of conforming to the rules is that it allows for the detection of the implicit rules of a phenomenon. For example, implicit learning research indicates that complex grammatical rules can be detected unconsciously [7], [8]. Therefore, an important question is, would UT facilitate the detection of complex rules more than CT would?

Recently, Mealor and Dienes (2012) combined UT and implicit learning research paradigms to investigate the impact of UT on artificial grammar learning [9]. A classic implicit learning paradigm consists of two stages: training and testing. In the training phase, participants are presented with random strings of letters, which are generated according to an artificial grammar rule. In the testing phase, participants are presented with new letter strings and asked to judge which of these follow the same grammatical rules as those presented during training. Mealor and Dienes (2012) extended this paradigm by introducing an intermediate phase between training and testing. In their study, participants in the UT group completed a 5-minute distractor task. Participants in the CT group spent the same 5-minute interval reflecting on the grammatical rule generated during training, and participants in the immediate decision group proceeded directly to testing immediately after the training phase. During the testing phase, participants were asked not only to judge whether each string conformed to grammatical rules but also to indicate whether their judgments were based on random selection, intuition, familiarity, rules, or recollection. Results showed that UT facilitated judgments based on random selection but did not facilitate the detection of rules based on intuition, familiarity, rules, or recollection.

It is important to note that in the study conducted by Mealor and Dienes (2012), the dependent variable was performance on a classification judgment (in the testing phase). Classification judgments are known to involve in not only the detection of rules, but also following rules [6], [10]. However, UT does not facilitate following rules. Moreover, in classic UT paradigms, participants were asked to either choose the right option explicitly or indicate their attitude toward each option. Therefore, the use of measures involving in following rules may affect observations concerning the role of UT in decision making. More importantly, the study used a learning task. Participants were only offered single-type information (many letter strings generated according to an artificial grammar rule), whereas classic UT paradigms participants were presented with several objects (four different cars), each with a different set of a positive or negative attributes, and were thus required to consider multiple variables when making their decisions. Compared to learning tasks involving single-type information, decision-making tasks with multiple variables require participants to not only integrate the information of each variable but also comparatively analyze the information between different variables. Therefore, we were interested in whether UT would facilitate the detection of rules during a complex decision-making task rather than a learning task.

In the present study, we presented two types of letter strings to participants: one conformed to a grammatical rule while the other did not. The participants were then assigned to one of three groups: CT, UT, or immediate decision. The experimental task required that participants choose which type of letter strings conformed to the established grammatical rule. If UT facilitates the detection of rules, accuracy in choosing the letter string that conformed to grammatical rules would be higher in the UT group than the CT and immediate decision groups.

## Methods

### Participants and Design

Participants were 120 Chinese undergraduate students (57 men and 63 women), and all were compensated for their participation. The students were randomly assigned into one of three groups: (1) UT (19 men and 21 women), (2) CT (20 men and 20 women), and (3) immediate decision (18 men and 22 women).

### Materials and Procedure

Grammar used in this study was taken directly from a study conducted by Dienes et al. [11]. Forty-eight letter strings (each 4–6 letters long) were generated, among which, 24 letter strings conformed to the grammatical rules while the other 24 did not. These ungrammatical letter strings were constructed via switching one letter of a grammatical string to ungrammatical one. To aid discrimination between the two types of letter string, the letter strings were composed of two groups of different letters. Half of the participants were presented with grammatical letter strings composed of the letters M, V, X, R, and T and ungrammatical strings composed of the letters W, S, N, P, and Z. The other half were presented with grammatical strings composed of the letters W, S, N, P, and Z and ungrammatical strings composed of the letters M, V, X, R, and T. In addition, the letter strings were presented in two colors: blue and green. Half of the participants were presented with grammatical letter strings composed of blue letters and ungrammatical strings composed of green letters. The other half were presented with grammatical letter strings composed of green letters and ungrammatical strings composed of blue letters. The types of letters and colors were counterbalanced across participants.

Before the experiment, participants were told that the study would involve decision-making tasks, and that they would see two types of letter strings containing different letters (M, V, X, R, T or W, S, N, P, Z) and colors (blue or green). They were also instructed to try their best to give an overall impression of each type of letter string. During the encoding phase, the two letter sets were presented consecutively and in counterbalanced order. Half of the participants were presented with grammatical and then ungrammatical letter strings, while the other half were presented strings in the opposite order. Twenty-four strings of each type were presented in random order and the duration of each presentation was 6 s.

Following presentation of the stimuli, all participants were informed that one of the two types of strings conformed to grammatical rules, and that they would be required to pick out this type of letter string. Subsequently, the participants were randomly assigned to one of three groups. Participants in the CT group were asked to think about the presented letter strings' characteristics (e.g., how the strings began or finished) for 5 minutes and were allowed to record their thinking process on a piece of paper on the desk. Two groups of letters (M, V, X, R, T and W, S, N, P, Z) were presented on a computer screen. The colors of the letters were exactly the same as those presented earlier in the session. Participants in the UT group were asked to complete a 2-back task for 5 minutes, to prevent them from consciously thinking about the letter strings [12]. Participants in the immediate decision group were asked to progress directly from the encoding phase to the testing phase.

During the testing phase, two types of letter string composed of two groups of letters (M, V, X, R, T and W, S, N, P, Z) were presented on the computer screen. The colors of the letters were the same as those presented earlier. Participants were then asked to choose the type of letter string that conformed to the grammatical rules. In addition, to control the influence of colors on the experimental results, we also asked the participants to rate their preference for the two colors on a 7-point scale (the higher the number, the more favorable the rating).

### Ethics Statement

The study was approved by the Institutional Review Board of the Department of Psychology at Northwest Normal University. All participants gave written consent prior to testing.

## Results

We calculated the accuracy of participants' identification of grammatical strings. As expected, participants in the UT group had the highest accuracy rate (70%), whereas the accuracy rates for the CT group (45%) and the immediate decision group (48%) were comparatively lower. Only the UT performed at a level greater than chance, χ^2^(1, N = 40) * = *6.40, *p*<.05. Further analysis revealed that participants in the UT group had higher accuracy rates than those in the CT group, χ^2^(1, N = 80)  = 5.11, *p*<.05, and the immediate decision group, χ^2^(1, N = 80) * = *4.18, *p*<.05. The accuracy scores of the CT and immediate decision groups did not differ significantly, χ^2^(1, N = 80) *<*1, *p* = .82. These results appear to demonstrate that following the presentation of the letter strings, UT facilitated the detection of complex rules to a greater extent than did either CT or immediate decision making (no delay between presentation and decision testing).

To exclude the effect of color on this study, we also analyzed the participants' color preference (for blue or green) according to thought type (UT, CT, and immediate). The results revealed non-significant main effects of thought type, *F*(2, 117)  = .21, *p>*.05 and color, *F*(1, 117)  = 1.32, *p>*.05. Further, the interaction between thought type and color was also non-significant, *F*(2, 117)  = .61, *p>*.05. As displayed in Table 1 are the descriptive statistics of colours under different thought conditions. These findings indicate that the color of the letter strings did not affect the study results concerning decision making.

**Table 1:** The descriptive statistics of colours under different thought conditions.

	Blue		Green	
	*M*	*SD*	*M*	*SD*
conscious thought	4.90	1.31	5.15	1.33
Immediate decision	5.00	1.30	4.95	1.51
Unconscious thought	4.85	1.41	5.35	1.59

## Discussion

The aim of the present study was to investigate whether UT could facilitate the detection of complex rules. We chose strings of letters as the experimental stimuli and compared participants' accuracy in detecting grammatical rules under conditions of CT, UT, and immediate decision making. We found that participants in the UT group identified the correct grammatical strings more accurately than participants in either the CT or immediate decision groups. This finding indicates that UT is beneficial to the detection of rules.

Our results have generated new evidence in support of UTT. An important finding from previous research on UT is that it is more suitable than CT for solving complex rather than simple decision-making tasks. In classic UT experiments, however, the complexity of the task is usually determined by the amount of information presented [13], [14]. Nonetheless, the level of complexity of a stimulus also includes the complexity of the rule that generated the stimulus information. The present study extends previous findings by showing that UT is indeed beneficial in solving decision-making tasks that have complex rules.

Our study has also expanded on the study conducted by Mealor and Dienes (2012), in which the dependent measure was a classification judgment (in the testing phase). In contrast, in our study, participants were merely asked to directly report which type of letter strings was more conforming to grammatical rules. This method would be more sensitive to measure the effects of UT than classification judgment. More importantly, the present study demonstrated that UT is important for the detection of rules in complex decision-making tasks. In future research, we should adopt the method used by Mealor and Dienes (2012), in which participants were asked to report whether their choice was based on random selection, intuition, familiarity, rules or recollection. Through this method, we will judge whether they make a decision according to the conscious or unconscious structural and judgment knowledge.

We suggest that our results may have been caused by UT organizing information in memory more efficiently. Studies have shown that UT may actively integrate and organize inforation in memory. Dijksterhuis (2004) indicated that UT made representations of information in memory more polarized and integrated. More importantly, Abadie, Waroquier, and Terrier (2013) demonstrated that the process of UT integrating information was gist-based, not verbatim-based [15]. If letter string rules are to be detected, the common characteristics of each type will be integrated while individual characteristics of letter strings will be ignored. Therefore, gist-based process facilitates the detection of rules. However, how does UT exactly distinguish grammatical and ungrammatical letter strings? In the current experiment, the ungrammatical letter strings are formed by switching one letter of grammatical ones, and the switched letter is always random, and not systematic. The other transitions in the sequence are grammatical. Considering the grammatical letter string is MTTTTV, and every transition between each letter in the sequence is grammatical. Then we switch one letter T to X, to form an ungrammatical letter string MTTXTV, and the other transitions are grammatical. It is possible that participants in the UT group are more sensitive to this transitions. This leads to the higher accuracy of UT choosing grammatical letter strings than that of CT and immediate groups [16], [17]. However, this hypothesis requires further confirmation through future experiments.
